# Clinical Utility of Quantitative Parameters of Salivary Gland Scintigraphy for Diagnosing Burning Mouth Syndrome

**DOI:** 10.3390/diagnostics12092256

**Published:** 2022-09-18

**Authors:** Hyung Kwon Byeon, Geum Cheol Jeong, Beomsoo Kim, Yeongrok Lee, Jae Hong Park, Sang Mi Lee

**Affiliations:** 1 Department of Otorhinolaryngology-Head and Neck Surgery, Soonchunhyang University Seoul Hospital, 59 Daesagwan-ro, Yongsan-gu, Seoul 04401, Korea; 2 Department of Nuclear Medicine, Soonchunhyang University Cheonan Hospital, 31, Suncheonhyang 6-gil, Dongnam-gu, Cheonan 31151, Korea; 3 Department of Otorhinolaryngology-Head and Neck Surgery, Soonchunhyang University Cheonan Hospital, 31, Suncheonhyang 6-gil, Dongnam-gu, Cheonan 31151, Korea

**Keywords:** burning mouth syndrome, radionuclide imaging, salivary gland, xerostomia

## Abstract

Burning mouth syndrome (BMS) is a chronic disorder characterized by a burning sensation in the oral cavity, often accompanied by xerostomia, with no relevant clinical or laboratory findings. This study aimed to investigate diagnostic values of quantitative parameters of salivary gland scintigraphy for BMS in patients with xerostomia. A total of 164 patients who underwent salivary gland scintigraphy for the workup of xerostomia were retrospectively reviewed. All patients were classified into patient groups with primary BMS, secondary BMS, and non-specific xerostomia. From salivary gland scintigraphy, 22 quantitative parameters were calculated and their diagnostic values were assessed based on the area under the receiver operating characteristic curve (AUC) values. Among salivary gland scintigraphy parameters, uptake speed in the left submandibular gland showed the highest AUC value (0.647) for detecting BMS and pre-stimulatory oral activity showed the highest AUC value (0.710) for detecting primary BMS. A salivary gland scintigraphy scoring system based on these two parameters further enhanced the diagnostic ability, demonstrating AUC values of 0.731 for BMS and 0.782 for primary BMS. These results suggest a potential diagnostic value of the quantitative parameters of salivary gland scintigraphy for detecting BMS in patients with xerostomia.

## 1. Introduction

Burning mouth syndrome (BMS) is a chronic pain syndrome characterized by a constant painful burning sensation of the oral mucosa, including the tongue, palate, and lip, in the absence of abnormal clinical and laboratory findings [1,2]. The prevalence of BMS in the general population ranges between 0.7% and 5.0%; BMS predominantly affects middle-aged and elderly women [1,3]. BMS is classified as primary BMS if there is no identified causative factor or as secondary BMS if there are causative factors such as hematologic deficiencies, autoimmune disorders, thyroid disorders, diabetes mellitus, psychological disorders, and medications [1]. The exact pathophysiological mechanism of BMS is currently unknown [1,4]. Nevertheless, since previous studies have found subclinical trigeminal neuropathy, decreased epithelial nerve fibers in the oral cavity, and enhanced factors associated with neuropathic pain in patients with BMS, the condition has been suggested to be a neuropathic disorder caused by a dysfunction, rather than the direct damage, of the somatosensory nervous system [4,5,6,7]. Furthermore, age, gender, laryngopharyngeal reflux, hormonal factors, and microbiota have also been suggested as etiopathological factors of BMS [8]. In patients with BMS, xerostomia is the second most common symptom, with a prevalence of up to 66% [1,9]. Xerostomia in patients with BMS has usually been considered a perceived symptom related to somatosensory dysfunction, rather than actual hyposalivation [9]. However, several recent studies have demonstrated a reduced unstimulated salivary flow rate with different saliva characteristics and proteomic profiles in patients with BMS as compared with healthy subjects [10,11,12]. A recent systemic review study regarding salivary characteristics in patients with BMS has also suggested that BMS is associated with changes in salivary biomarkers of inflammation and oxidative stress [13].

Salivary gland scintigraphy using ^99m^Tc pertechnetate is an imaging examination that can evaluate the uptake and excretion of the salivary glands [14]. In previous studies, it demonstrated sufficient sensitivity to detect mild salivary gland dysfunction caused by damage to only 25% of the gland parenchyma [15]. Therefore, for several decades, salivary gland scintigraphy has been used for aiding the diagnosis of Sjögren’s syndrome and assessing salivary gland function in patients with xerostomia [16,17,18]. Furthermore, by performing dynamic salivary gland scintigraphy, various quantitative imaging parameters can be measured from each gland, enabling an objective and precise assessment of salivary gland function [14,18]. Considering that recent studies have demonstrated significant association of BMS with inflammatory salivary biomarkers, salivary gland scintigraphy findings might provide clues for understanding the pathophysiology of BMS, thereby aiding the diagnosis of this disease. However, currently, there are very few studies that have investigated salivary gland scintigraphy findings in patients with BMS [10,19,20]. Although a significant portion of patients with xerostomia were diagnosed with BMS, a previous study only compared salivary gland scintigraphy findings between BMS patients with and without hyposalivation, and the diagnostic ability of salivary gland scintigraphy for detecting BMS in patients with xerostomia has not been reported yet [10,20].

Therefore, the aim of the present study was to evaluate whether quantitative parameters of salivary gland scintigraphy had diagnostic potential for detecting BMS in patients with xerostomia.

## 2. Materials and Methods

### 2.1. Study Participants

Electronic medical records of 242 consecutive patients who underwent salivary gland scintigraphy for the diagnostic workup of xerostomia between January 2018 and June 2021 in Soonchunhyang University Cheonan Hospital were retrospectively reviewed. Among these patients, 78 patients who (1) had a history of treatment for head and neck cancer, (2) were diagnosed with rheumatic diseases including Sjögren’s syndrome, (3) were on medications that could cause hyposalivation including antipsychotics, antidepressants, anticholinergics, and antihistamines, and (4) had oral mucosal diseases on clinical examination were excluded from this study. A total of 164 patients with xerostomia were finally enrolled in the study. Based on their clinical diagnoses, enrolled patients were classified into three patient groups: primary BMS, secondary BMS, and non-specific xerostomia groups. The diagnosis of BMS was made in accordance with the criteria of the International Classification of Headaches as follows: an oral pain that (1) has a burning quality that is felt superficially in the oral mucosa and (2) recurs daily for >2 h per day for >3 months with (3) normal appearance of oral mucosa on physical examination [21]. Patients clinically diagnosed with BMS were further categorized into two groups based on the presence of causative factors: primary BMS, defined as BMS with no identified causative factor, and secondary BMS, defined as BMS with causative factors such as endocrine disorders, psychological disorders, and neuropathy [1]. All other patients not diagnosed with BMS were classified as a non-specific xerostomia group.

When calculating the sample size of this study, the prevalence of BMS among subjects with xerostomia was set to be 20% based on previous studies [20,22]. A minimum number of the sample size was determined to be 100 participants with 20 positive cases in order to obtain a sensitivity of 80% with a power of 80% and a confidence of 95% [23].

The present study was approved by the Institutional Review Board of Soonchunhyang University Cheonan Hospital and was conducted in accordance with the Declaration of Helsinki. Informed consent from the patients was waived by the Institutional Review Board owing to the retrospective nature of the study

### 2.2. Salivary Gland Scintigraphy

Salivary gland scintigraphy imaging was performed with a dual-head gamma camera (Infinia GP, GE Healthcare, Milwaukee, WI, USA) equipped with a low-energy, high-resolution collimator using a dynamic scan protocol. Patients were instructed to fast for 2 h before scanning. Imaging was performed in the supine position with a slight extension of the patient’s head, and the detector was positioned anteriorly. Immediately after an intravenous injection of 555 MBq of ^99m^Tc pertechnetate, dynamic salivary gland scintigraphy images were sequentially acquired at 30 s per frame for 30 min on a 64 × 64 matrix. At 20 min after injecting the radiotracer, 10 mL of lemon juice was administered to stimulate salivary glands’ excretion.

### 2.3. Image Data Analysis

**Figure 1:**
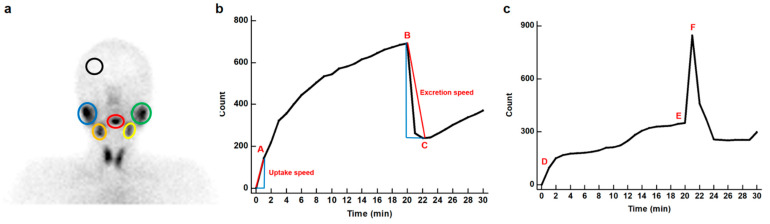
Examples of regions of interest (ROIs) on a salivary gland scintigraphy image (**a**) and time–activity curves of the salivary gland (**b**) and oral cavity (**c**) generated from salivary gland scintigraphy. (**a**) ROIs were manually drawn over each salivary gland (right parotid gland, blue oval; left parotid gland, green oval; right submandibular gland, orange oval; left submandibular gland, yellow oval), oral cavity (red oval), and background (black circle). (**b**) From the time–activity curve of the salivary gland, count at the end of the initial vascular perfusion phase (A), maximum count before stimulation (B), and minimum count after stimulation (C) were measured for calculating uptake ratio, maximum accumulation, and ejection fraction. Uptake speed was defined as count increment per second in the initial vascular perfusion phase, and excretion speed was defined as count decrement per second in the excretion phase. (**c**) From the time–activity curve of the oral cavity, count at the end of the initial vascular perfusion phase (D), maximum count before stimulation (E), and maximum count after stimulation (F) were measured for calculating pre-stimulatory oral activity and post-stimulatory oral activity.

For the quantitative analysis of salivary gland scintigraphy, the uptake of bilateral parotid and submandibular glands, background, and oral cavity were measured for each patient. For the salivary gland uptake measurement, an oval-shaped region of interest (ROI) was manually drawn over each parotid and submandibular gland (Figure 1a). Background uptake was measured by placing an ROI over the skull vault. Oral cavity uptake was measured by drawing an ROI over the oral cavity while avoiding the activity of the nose and submandibular glands. Based on the counts of those ROIs, time–activity curves for bilateral parotid and submandibular glands, background, and oral cavity were generated.

From the time–activity curves, a total of 20 quantitative parameters for salivary glands and 2 quantitative parameters for the oral cavity were calculated for each patient based on the methods used in previous studies [16,24,25]. For each salivary gland, five quantitative imaging indices (uptake ratio, maximum accumulation, ejection fraction, uptake speed, and excretion speed) were calculated using the following formulae (Figure 1b):

(Uptake ratio) = (maximum count before stimulation)/(background activity)

(Maximum accumulation) = [(maximum count before stimulation) − (count at the end of the initial vascular perfusion phase or if unclear, at 1 min)]/(maximum count before stimulation) × 100

(Ejection fraction) = [(maximum count before stimulation) − (minimum count after stimulation)]/(maximum count before stimulation) × 100

(Uptake speed) = (count increment per second in the initial vascular perfusion phase or, if unclear, in the first 60 s)

(Excretion speed) = (count decrement per second in the excretion phase)

Uptake ratio and maximum accumulation refer to the amount of radioactivity accumulated in the salivary glands, and ejection fraction reflects the degree of stimulated excretion of the salivary glands [16,25]. Uptake speed and excretion speed refer to the rates of accumulation and stimulated excretion of salivary glands, respectively [24,25]. Regarding radioactivity in the oral cavity, pre-stimulatory oral activity and post-stimulatory oral activity were calculated with the following formulae (Figure 1c):

(Pre-stimulatory oral activity) = [(maximum count before stimulation) − (count at the end of the initial vascular perfusion phase or if unclear, at 1 min)]/(maximum count before stimulation) × 100

(Post-stimulatory oral activity) = [(maximum count after stimulation) − (count at the end of the initial vascular perfusion phase or, if unclear, at 1 min)]/(maximum count after stimulation) × 100

Pre-stimulatory oral activity and post-stimulatory oral activity reflect the quantities of unstimulated spontaneous salivary excretion and stimulated salivary excretion, respectively [26].

### 2.4. Statistical Analysis

Comparisons of 22 salivary gland scintigraphy parameters among patients with primary BMS, secondary BMS, and non-specific xerostomia were performed using the Kruskal–Wallis test. For parameters that showed statistical significance in the Kruskal–Wallis test, a post hoc analysis using Dunn’s test was further performed. The diagnostic abilities of salivary gland scintigraphy for detecting BMS and primary BMS were assessed based on the area under the receiver operating characteristic (ROC) curve (AUC) values. A bootstrap method with 1000 iterations was used to estimate the 95% confidence interval (CI) for the AUC value of each quantitative parameter. Optimal cutoff values of salivary gland scintigraphy parameters were determined with the Youden index. With defined cutoff values, the sensitivity, specificity, positive predictive value, and negative predictive value of the parameters for detecting BMS and primary BMS were calculated. Using the parameters that showed the highest AUC values, a salivary gland scintigraphy scoring system for detecting BMS and primary BMS was devised. In each patient, a score of 1 was assigned for the parameters that showed lower values than the optimal cutoff values and a score of 0 was assigned for the parameters that showed equal or higher values than the cutoff values. The scoring system was based on the summation of scores of the included parameters, and the diagnostic ability of the scoring system for detecting BMS and primary BMS was evaluated by calculating AUC values. Statistical analyses were performed using MedCalc Statistical Software version 20.110 (MedCalc Software Ltd., Ostend, Belgium). A *p*-value of <0.05 was regarded as statistically significant.

## 3. Results

### 3.1. Participants

Of 164 enrolled patients, 29 patients (17.7%) were clinically diagnosed with primary BMS and 30 patients (18.3%) were diagnosed with secondary BMS. The remaining 105 patients (64.0%) were classified as the non-specific xerostomia group. The primary BMS group consisted of 6 men (20.7%) and 23 women (79.3%) and the median age was 62 years (range, 36–84 years). The secondary BMS group consisted of 9 men (30.0%) and 21 women (70.0%) and the median age was 63 years (range, 37–93 years). Further, the non-specific xerostomia group consisted of 32 men (30.5%) and 73 women (69.5%) and the median age was 59 years (range, 13–84 years). There were no significant differences among three patient groups with regard to age (*p* = 0.282) and sex (*p* = 0.578).

### 3.2. Comparison of Salivary Gland Scintigraphy Parameters

**Figure 2:**
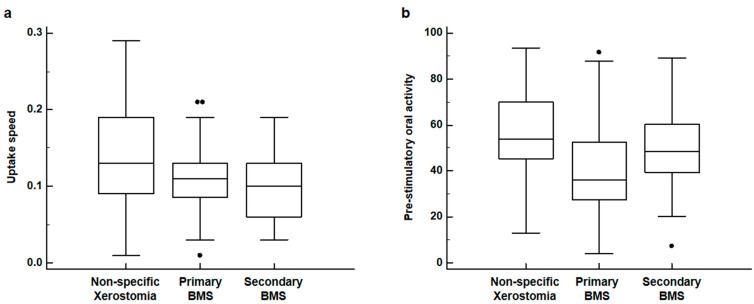
Distribution of uptake speed of left submandibular gland (**a**) and pre-stimulatory oral activity (**b**) in patients with non-specific xerostomia, primary burning mouth syndrome (BMS), and secondary BMS (black dots: an outside value which is larger than the 75 percentile value plus 1.5 times the interquartile range or smaller than the 25 percentile value minus 1.5 times the interquartile range).

Comparative analyses of salivary gland scintigraphy parameters among primary BMS, secondary BMS, and non-specific xerostomia groups are shown in Table 1. On the Kruskal–Wallis test, significant differences in the uptake speed of the left submandibular gland (*p* = 0.024) and pre-stimulatory oral activity (*p* < 0.001) were found among the three patient groups, while none of the other salivary gland scintigraphy parameters showed any significant differences (*p* > 0.05). On post hoc comparison using Dunn’s test, patients in both the primary and secondary BMS groups showed significantly lower values of uptake speed in the left submandibular gland than those in the non-specific xerostomia group (*p* < 0.05), whereas there was no significant difference in uptake speed between the primary and secondary BMS groups (*p* > 0.05) (Figure 2a). With respect to pre-stimulatory oral activity, patients in the primary BMS group showed significantly lower values than those with non-specific xerostomia (*p* < 0.05), while no significant differences were observed between patients with primary and secondary BMS or between patients with secondary BMS and non-specific xerostomia (*p* > 0.05) (Figure 2b).

### 3.3. Diagnostic Ability of Salivary Gland Scintigraphy Parameters

**Figure 3:**
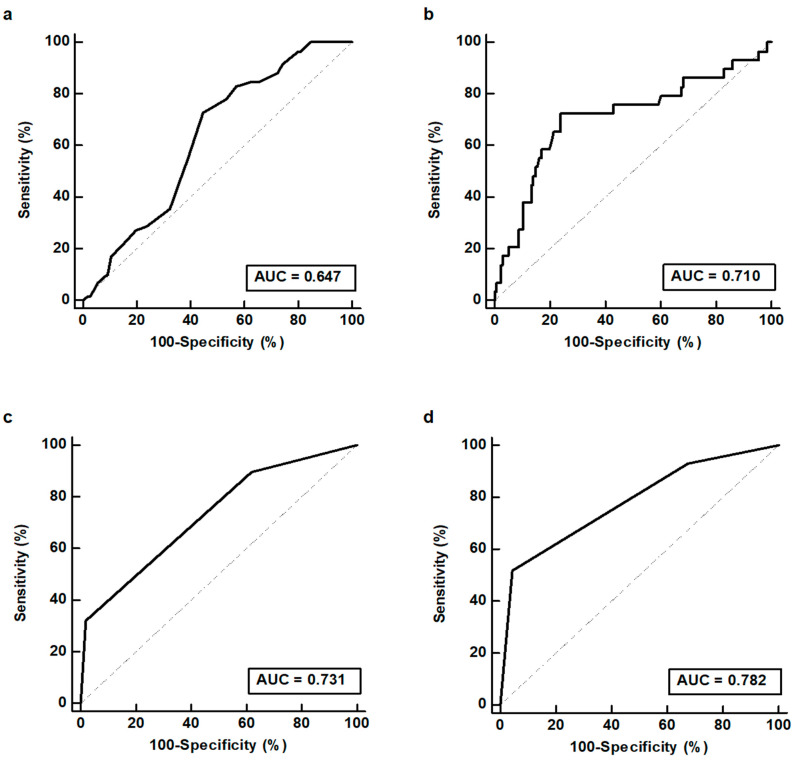
Receiver operating characteristic curves for uptake speed of left submandibular gland (**a**) and salivary gland scintigraphy scoring system (**c**) for detecting burning mouth syndrome (BMS). Receiver operating characteristic curves for pre-stimulatory oral activity (**b**) and salivary gland scintigraphy scoring system (**d**) for detecting primary BMS.

The diagnostic abilities of salivary gland scintigraphy for detecting BMS (both primary and secondary forms) and primary BMS were assessed based on ROC curve analysis (Table 2). Among salivary gland scintigraphy parameters, the uptake speed in the left submandibular gland showed the highest AUC value (0.647; 95% CI, 0.568–0.711) for detecting patients with BMS (Figure 3a). Using an optimal cutoff value of 0.14, the uptake speed in the left submandibular gland showed a sensitivity of 83.1% (95% CI, 71.0–91.6%), a specificity of 42.9% (95% CI, 33.2–52.9%), a positive predictive value of 45.0% (95% CI, 40.0–50.0%), and a negative predictive value of 81.8% (95% CI, 71.0–89.2%). For detecting patients with primary BMS, pre-stimulatory oral activity revealed the highest AUC value (0.710; 95% CI, 0.634–0.778) among parameters (Figure 3b). Using a cutoff value of 41.5, pre-stimulatory oral activity showed a sensitivity of 72.4% (95% CI, 52.8–87.3%), a specificity of 76.3% (95% CI, 68.2–83.2%), a positive predictive value of 39.6% (95% CI, 31.0–48.9%), and a negative predictive value of 92.8% (95% CI, 87.6–95.9%).

### 3.4. Salivary Gland Scintigraphy Scoring System

Using the uptake speed in the left submandibular gland and pre-stimulatory oral activity, a salivary gland scintigraphy scoring system for detecting BMS and primary BMS was made. In the scoring system, patients who showed low values of those parameters (uptake speed of the left submandibular gland < 0.14 or pre-stimulatory oral activity < 41.5) were assigned a score of 1 and those who showed high values (uptake speed of the left submandibular gland ≥ 0.14 or pre-stimulatory oral activity ≥ 41.5) were assigned a score of 0. Consequently, the summed score of patients ranged from score 0 to score 2. This scoring system further enhanced the diagnostic ability of salivary gland scintigraphy for detecting BMS and primary BMS (Table 3). Among 21 patients with a score of 2 (low values of both parameters), 19 (90.5%) and 15 (71.4%) were diagnosed with BMS and primary BMS, respectively. Meanwhile, only 13.0% and 4.3% of 46 patients with a score of 0 (high values of both parameters) were diagnosed with BMS and primary BMS, respectively. The salivary gland scintigraphy scoring system demonstrated AUC values of 0.731 (95% CI, 0.656–0.797) for detecting BMS (Figure 3c) and 0.782 (95% CI, 0.711–0.843) for detecting primary BMS (Figure 3d).

## 4. Discussion

Although the pathophysiology of BMS is largely unknown, one of the suggested mechanisms involves taste and sensory system interactions [27]. In addition to pain and a burning sensation in the oral cavity, reduced or distorted taste perception is also a very common symptom in patients with BMS, which has been found in approximately two-thirds of patients [28]. In a previous study, dysfunction of the chorda tympani, a branch of the facial nerve that innervates taste sensation of the anterior two-thirds of the tongue, was found in 82% of patients with BMS, suggesting that alterations of taste sensation in BMS could be a result of damage to the chorda tympani [29]. Through central nervous system interactions, damage in the taste nerve is known to disinhibit undamaged sensory nerves [30]. In other words, damage to the chorda tympani can lead to loss of inhibition of the trigeminal nerve, which innervates sensations of the anterior two-thirds of the tongue and oral mucosa, and these interactions can result in both pain and taste disturbance in patients with BMS [27,30].

Along with taste sensation, the chorda tympani can also mediate the secretion of the submandibular and sublingual salivary glands, which raises the hypothesis that xerostomia in patients with BMS could be due to salivary gland hypofunction induced by nerve damage [31,32]. However, several previous studies argued that xerostomia in patients with BMS is merely a subjective symptom or caused by side effects of medications, rather than by actual alterations to salivary gland function [3]. In contrast, other recent studies demonstrated that patients with BMS have significantly lower unstimulated salivary flow rates than control subjects regardless of medications, whereas no significant difference was found in stimulated salivary flow rates between them [10,11,32,33]. Unstimulated salivary flow reflects the basal level of salivary flow in a resting status, and approximately 70% of unstimulated saliva derives from the submandibular and sublingual glands, while most of stimulatory saliva is excreted from the parotid glands [34]. Therefore, it has been hypothesized that a decreased unstimulated salivary flow rate implies that the function of the submandibular and sublingual glands at a resting status could be impaired in patients with BMS [10,32]. The results of the present study also support this hypothesis. In this study, patients with BMS showed significantly lower values of uptake speed in the left submandibular gland and pre-stimulatory oral activity on salivary gland scintigraphy than those with non-specific xerostomia. Considering that pre-stimulatory oral activity reflects the quantity of unstimulated salivary excretion in the oral cavity, the results of the present study indicate that uptake and excretion functions of the submandibular glands at a resting status were reduced, which could be imaging evidence for submandibular gland hypofunction in patients with BMS. The saliva excreted from the submandibular glands at a resting status plays a significant role in the lubrication and oral mucosal protection, and oral dryness has been found to increase pain sensitivity in the oral mucosa [35,36]. Therefore, dysfunction of the submandibular glands could contribute to the aggravation of a painful sensation in the oral mucosa as well as xerostomia. In contrast, all parameters of the parotid gland and parameters concerning stimulatory salivary excretion, such as ejection fraction, excretion speed, and post-stimulatory oral activity, did not show any significant differences between patients with BMS and those with non-specific xerostomia. These findings are in agreement with the results of previous studies [10,32,33], suggesting that stimulatory salivary excretion is preserved in patients with BMS. Accordingly, in future studies of BMS, the unstimulated salivary excretion function at a resting status should be the main focus.

In the literature, few studies have evaluated salivary gland scintigraphy findings in patients with BMS, and the diagnostic value of salivary gland scintigraphy for BMS has yet to be assessed [10,19,20]. In a previous study, salivary gland scintigraphy parameters were measured from 33 patients with primary BMS and were compared between BMS patients with and without hyposalivation [10]. In that study, only two quantitative parameters, maximum accumulation and ejection fraction, were calculated from salivary gland scintigraphy, and the results revealed no significant differences in those two parameters between the patient groups [10]. From these insignificant findings, that study suggested that hyposalivation in primary BMS might not be a result of salivary gland dysfunction [10]. However, when taken together with the results of this study, salivary gland scintigraphy parameters of maximum uptake and stimulatory excretory function of the salivary glands might have a limited value for clinical use in patients with BMS. In other studies, a primary aim was to assess the clinical value of salivary gland scintigraphy in differential diagnosis of Sjögren’s syndrome from other diseases with xerostomia including BMS; the findings of salivary gland scintigraphy in patients with BMS were not the main focus [19,20]. On the other hand, in the present study, patients with a history of treatment for head and neck cancer and rheumatic disease, which could lead to severe dysfunction of salivary glands, were excluded; we investigated whether quantitative parameters of salivary gland scintigraphy had a clinical role for diagnosing BMS in patients with xerostomia. The results of the present study demonstrated that uptake speed of the left submandibular gland had a high sensitivity (83.1%) and a negative predictive value (81.8%) for diagnosing BMS and that pre-stimulatory oral activity had a high negative predictive value (92.8%) for diagnosing primary BMS with moderate sensitivity and specificity. Moreover, a scoring system that combined those two parameters could further stratify the probability of BMS and primary BMS. Among patients who had low values of both parameters, 90.5% and 71.4% were diagnosed with BMS and primary BMS, respectively, whereas only 13.0% and 4.3% of patients with high values of both parameters were diagnosed with BMS and primary BMS, respectively. Although the diagnosis of BMS is based mainly on the history and physical examination of patients after excluding other diseases [1,4], the results of the present study suggest that quantitative parameters of salivary gland scintigraphy might provide helpful information for diagnosing BMS in patients with xerostomia.

There are several limitations in the present study. First, this study enrolled a small number of patients from a single medical center. Hence, there might be a potential bias. Second, since this study was retrospectively performed without a randomization process, further external validation of the results is needed. Third, only the differences in quantitative parameters of salivary gland scintigraphy between patients with BMS and non-specific xerostomia were evaluated, and there was no reference standard to define salivary gland dysfunction in the enrolled patients. Fourth, because only patients with xerostomia were included in the study, salivary gland scintigraphy findings of BMS patients without xerostomia could not be evaluated, which might limit the general application of findings of this study to patients with BMS. Finally, to provide a relevant basis for the use of salivary gland scintigraphy in patients with BMS, the underlying mechanism of salivary gland scintigraphy findings also needs further investigation.

## 5. Conclusions

In the present study, patients with BMS showed significantly lower values of uptake speed of the left submandibular gland and pre-stimulatory oral activity on salivary gland scintigraphy than those with non-specific xerostomia. Combining those two parameters provided a significant diagnostic value for BMS, showing AUC values of 0.731 for detecting BMS and 0.782 for detecting primary BMS. The present results might be considered imaging evidence for dysfunctional unstimulated salivary excretion of the submandibular glands in patients with BMS and suggests that salivary gland scintigraphy might help diagnose BMS in patients with xerostomia. However, because the present study enrolled only patients with xerostomia in a retrospective manner, further validation of the clinical use of salivary gland scintigraphy in patients with BMS is necessary.

## Figures and Tables

**Table 1:** Comparisons of salivary gland scintigraphy parameters among patients with primary BMS, secondary BMS, and non-specific xerostomia.

Parameters		Primary BMS (*n* = 29)	Secondary BMS (*n* = 30)	Non-Specific Xerostomia (*n* = 105)	*p*-Value *
Uptake ratio	Right parotid	5.00 (3.45–5.74)	4.29 (3.75–4.73)	4.64 (3.78–5.85)	0.528
	Left parotid	4.78 (3.81–5.82)	4.72 (4.16–5.13)	5.05 (3.87–5.95)	0.822
	Right submandibular	3.52 (2.97–4.30)	3.49 (3.06–3.93)	3.88 (3.08–4.43)	0.360
	Left submandibular	3.58 (2.95–4.25)	3.56 (3.17–3.83)	3.79 (3.05–4.40)	0.493
Maximum accumulation	Right parotid	76.3 (68.1–82.3)	80.6 (72.9–88.1)	76.6 (69.9–84.9)	0.233
	Left parotid	78.0 (66.8–84.8)	81.3 (72.7–87.5)	79.3 (68.9–86.2)	0.438
	Right submandibular	48.8 (39.8–66.0)	61.5 (48.3–74.0)	57.0 (38.5–71.0)	0.193
	Left submandibular	50.6 (40.3–67.2)	62.1 (49.6–77.8)	58.6 (40.0–72.5)	0.225
Ejection fraction	Right parotid	43.8 (24.6–47.4)	31.2 (23.3–36.8)	33.3 (21.6–46.3)	0.389
	Left parotid	39.4 (24.5–46.5)	32.3 (20.8–39.4)	31.2 (18.5–41.2)	0.345
	Right submandibular	19.8 (10.7–30.9)	21.7 (10.3–33.0)	26.3 (14.0–34.7)	0.165
	Left submandibular	18.5 (8.2–30.7)	19.2 (8.5–27.4)	25.0 (13.7–31.9)	0.195
Uptake speed	Right parotid	0.25 (0.14–0.29)	0.17 (0.10–0.22)	0.21 (0.13–0.31)	0.123
	Left parotid	0.19 (0.14–0.28)	0.17 (0.10–0.24)	0.20 (0.10–0.30)	0.420
	Right submandibular	0.13 (0.11–0.17)	0.12 (0.07–0.13)	0.14 (0.09–0.20)	0.138
	Left submandibular	0.11 (0.08–0.13)	0.10 (0.06–0.13)	0.13 (0.09–0.19)	0.024
Excretion speed	Right parotid	0.51 (0.22–0.72)	0.38 (0.21–0.57)	0.42 (0.23–0.73)	0.692
	Left parotid	0.50 (0.25–0.64)	0.38 (0.23–0.59)	0.36 (0.23–0.61)	0.678
	Right submandibular	0.27 (0.09–0.50)	0.37 (0.21–0.59)	0.44 (0.18–0.64)	0.159
	Left submandibular	0.26 (0.08–0.49)	0.32 (0.11–0.47)	0.35 (0.18–0.66)	0.294
Pre-stimulatory oral activity		36.0 (27.3–52.7)	48.6 (39.2–60.3)	53.8 (45.2–69.9)	<0.001
Post-stimulatory oral activity		59.2 (46.4–65.7)	63.0 (54.4–72.3)	63.5 (49.1–75.6)	0.238

Expressed in median value (25th–75th percentiles). * Results of Kruskal–Wallis test.

**Table 2:** Diagnostic utility of salivary gland scintigraphy parameters for detecting BMS and primary BMS.

Parameters		BMS		Primary BMS	
		AUC	95% CI	AUC	95% CI
Uptake ratio	Right parotid	0.532	0.452–0.610	0.518	0.438–0.596
	Left parotid	0.527	0.447–0.605	0.509	0.429–0.587
	Right submandibular	0.566	0.486–0.643	0.536	0.457–0.614
	Left submandibular	0.549	0.470–0.627	0.512	0.433–0.591
Maximum accumulation	Right parotid	0.522	0.442–0.600	0.560	0.480–0.637
	Left parotid	0.524	0.445–0.602	0.536	0.457–0.614
	Right submandibular	0.520	0.441–0.599	0.567	0.487–0.644
	Left submandibular	0.529	0.449–0.607	0.553	0.473–0.630
Ejection fraction	Right parotid	0.504	0.425–0.583	0.567	0.488–0.644
	Left parotid	0.548	0.469–0.626	0.566	0.487–0.622
	Right submandibular	0.585	0.505–0.661	0.575	0.496–0.651
	Left submandibular	0.584	0.505–0.661	0.553	0.473–0.630
Uptake speed	Right parotid	0.554	0.474–0.631	0.537	0.458–0.615
	Left parotid	0.542	0.462–0.620	0.513	0.434–0.592
	Right submandibular	0.567	0.487–0.644	0.513	0.433–0.591
	Left submandibular	0.647	0.568–0.711	0.586	0.507–0.663
Excretion speed	Right parotid	0.517	0.438–0.596	0.523	0.444–0.601
	Left parotid	0.530	0.451–0.609	0.552	0.472–0.629
	Right submandibular	0.580	0.500–0.656	0.584	0.505–0.660
	Left submandibular	0.574	0.494–0.650	0.561	0.481–0.638
Pre-stimulatory oral activity		0.627	0.548–0.710	0.710	0.634–0.778
Post-stimulatory oral activity		0.537	0.458–0.615	0.599	0.519–0.674

AUC, area under the receiver operating characteristic curve; BMS, burning mouth syndrome; CI, confidence interval.

**Table 3:** Detection accuracy of the salivary gland scintigraphy scoring system for BMS and primary BMS.

Scoring System	BMS		Primary BMS		
	BMS (+) *(n* = 59)	BMS (−) * (n = 105)	Primary BMS (+) (*n* = 29)	Primary BMS (−) † (*n* = 135)	Total
0	6 (13.0%)	40 (87.0%)	2 (4.3%)	44 (95.7%)	46 (100.0%)
1	34 (35.1%)	63 (64.9%)	12 (12.4%)	85 (87.6%)	97 (100.0%)
2	19 (90.5%)	2 (9.5%)	15 (71.4%)	6 (28.6%)	21 (100.0%)

BMS, burning mouth syndrome. * Non-specific xerostomia. † Secondary BMS and non-specific xerostomia.

## Data Availability

The datasets generated during and/or analyzed during the current study are available from the corresponding authors on reasonable request.
